# A practical guide to delivering personalisation. Person-centered practice in health and social care

**Published:** 2013-06-07

**Authors:** Michel Botbol

**Affiliations:** WPA French Member Societies Association, Co-Chair of the WPA Section on Psychoanalysis in Psychiatry. E-mail: botbolmichel@orange.fr

Person centeredness appears increasingly as a major concept for the health systems in most parts of the world. But its definition and the ways to implement it are still too vague to warrant good enough programs and policies to reach this ambition. The aim of “A practical guide to delivering personalization” is obviously to fill in this gap as indicated by the book’s subtitle: “person-centered practice in health and social care”.

Very well documented and supported by the strong commitments of its authors to advocate the person-centered perspectives, the book is divided into four parts presenting the basic principles required to deliver personalization through person-centered interventions. Questioning person-centered practice (why this, why now) part I defines clearly the concept of personalization, and, through an historical approach of person-centered planning and thinking, the context from which it is emerging. In this way it highlights the values underpinning a person centered approach (independence and rights, choice and control, focus on capacity, promoting inclusive communities) and the individual and institutional challenges it is currently facing to achieve these goals. Part II discusses the theoretical backgrounds of these challenges, insisting on what it considers a major one: how to find a practical way to build true change into the thinking on which health and social care providers ground their attention to a person’s needs. It builds its demonstration on the presentation of well illustrated “simple and practical person-centered clinical tools” to support the implementation of person centered thinking in professionals and services (for example, learning and understanding the balance between what is important to and for the person through various tools, such as good days, bad days, routines or enhancing choice and control through communication charts or decision-making profiles etc.). On these bases, part III shows how it is possible to really make the difference in various important fields including the care program approach in mental health services through person-centered care and treatment planning. The last part (part IV) goes further in the same direction applying the person-centered approach to various situations “from prevention to end of life”. In this ambitious journey the book avoids the pitfall to which such large range perspectives are frequently exposed: a mere listing of concepts weakly linked by general principles. Indeed, the authors carry out successfully their presentation of each of these applications as a way to enlighten, from various points of view, the concepts and practices exposed in the previous parts of the book. This last approach confirms the overall main achievement of this work: to show that more than anything else, person-centeredness is a practical issue: something to *do* rather than to merely proclaim.

In a context in which technical advances and economic pressures may lead one to forget the importance of many human values in the design of services, this practical approach of integrated care is surely a major contribution to bringing these values back to the center of service users’, stake holders’ and professionals’ attention.

